# Slow-Freezing Cryopreservation Ensures High Ovarian Tissue Quality Followed by In Vivo and In Vitro Methods and Is Safe for Fertility Preservation

**DOI:** 10.3390/medicina56100547

**Published:** 2020-10-19

**Authors:** Živilė Gudlevičienė, Kastytis Žilinskas, Gabrielis Kundrotas, Monika Grubliauskaitė, Daiva Baltriukienė, Virginija Bukelskienė

**Affiliations:** 1 Department of Biobank, National Cancer Institute, Santariskiu Str. 1, 08660 Vilnius, Lithuania; gabrielis.kundrotas@gmail.com (G.K.); monika.grubliauskaite@nvi.lt (M.G.); 2 Department of Oncogynecology, National Cancer Institute, Santariskiu Str. 1, 08660 Vilnius, Lithuania; kastytis.zilinskas@nvi.lt; 3 Department of Biological Models, Institute of Biochemistry, Life Sciences Center, Vilnius University, Sauletekio al. 7, 10257 Vilnius, Lithuania; daiva.baltriukiene@bchi.vu.lt (D.B.); virginija.bukelskiene@bchi.vu.lt (V.B.)

**Keywords:** ovarian tissue cryopreservation, young cancer patients, quality control, xenotransplantation, miRNA

## Abstract

*Background and objectives:* Cancer incidence is growing with younger patients diagnosed with this disease every year. Improved cancer diagnostics and treatment lead to better survival of cancer patients. However, after aggressive chemo- or radiotherapy, cancer survivors suffer from various degrees of subfertility or infertility. Several fertility preservation technologies have been developed for young cancer patients: cryopreservation of germ cells, embryos, or reproductive tissues. The best results have been shown by cryopreservation of sperm and embryos. Yet the success of using cryopreserved oocytes or reproductive tissues (ovarian and testicular) is still insufficient. Therefore, this study was designed to assess the vitality, viability, general quality, and safety of frozen–thawed human ovarian tissue for retransplantation using modern molecular tests. *Materials and Methods:* The new miRNA array test was used to evaluate miRNA expression in thawed ovarian tissue in combination with standard xenotransplantation and pathological examination of microslides. *Results:* Our results demonstrated that slow freezing is an efficient way (80%) to cryopreserve ovarian tissue with no structural damage afterwards. We have shown that xenotransplantation into immunodeficient mice, histology, and immunohistochemistry could be potentially replaced by more recent molecular methods. *Conclusions:* The latter method has shown that altered expression of miRNAs might be used as identifiers of normal/damaged tissue after further analysis. Newer, safer, and more specific approaches need to be developed in order to eliminate the risk of disease reoccurrence.

## 1. Introduction

Thanks to advancements in cancer diagnostics and treatment protocols, female survival rates have increased in recent decades, especially among young patients. However, due to aggressive treatment, usually systemic chemotherapy and radiotherapy, the probability of successful reproduction is reduced dramatically [1]. Consequently, patients within their reproductive years or adolescent patients should be referred to a specialist to discuss fertility preservation options [2,3].

To date, there have been several strategies for fertility preservation to prolong the ability to conceive for female cancer survivors. These involve cryopreservation of oocytes, embryos, or ovarian tissue [2,4,5]. Even though ovarian tissue cryopreservation (OTC) is considered experimental, this strategy might be the only option for pre-pubescent girls and patients who need immediate gonadotoxic treatment or for whom hormonal stimulation treatment is contraindicated [3,6]. Other more recently developed experimental options are the following: immature oocyte cryopreservation for pre-pubescent female patients [5], though no successful pregnancies have been reported [7]; creation of a personalized artificial ovary [8] or ovarian suppression for post-pubescent female patients [4]. In comparison with the cryopreservation of oocytes and embryos, OTC can retain full organ function with the production of hormones, followed by the first spontaneous pregnancy [9,10]. There have been more than 100 successful births reported to date using the reimplantation of thawed ovarian tissue and the number is increasing every year [10].

Cryopreservation of ovarian tissue (OT) can be performed by conventional slow freezing or vitrification [11,12,13,14,15]. However, there is a probability of reintroducing malignant cells after autotransplantation to a cancer survivor [7]. The most common method to verify this is a xenotransplantation experiment. Many studies have been conducted to assess tissue vitality after thawing by using more recent and less time-consuming techniques. These practices include histological and immunohistochemical evaluations for: (1) structural, functional, and DNA integrity; (2) malignant cell contamination; and polymerase chain reaction (PCR) for the detection of molecular tumor markers [16,17,18]. Moreover, currently, disparities in steroid production and altered expression of specific microRNAs (miRNAs), e.g., miRNA–193b, miRNA–320A, and miRNA–24, after cryopreservation have been associated with failure of ovarian tissue functioning and folliculogenesis [19]. MicroRNAs are a class of small non–coding RNA molecules that have been shown to be potential biomarkers for different cancers and prognostics [20] and are involved in gametogenesis, embryo development, and fertility [21,22].

In this study, we investigate whether a time-consuming and expensive xenotransplantion method could be replaced by shorter quality control tests in order to identify contamination with cancer cells, viability, and structural integrity of frozen–thawed ovarian tissue fragments before retransplantation.

## 2. Material and Methods

### 2.1. Patients

From 2014 to 2018, 28 young female cancer patients from the National Cancer Institute (NCI, Vilnius, Lithuania) were included in the “*Fertility preservation program for cancer patients*”. All women signed patient information and informed consent. The study protocol, patient information and informed consent were approved by the Vilnius Regional Biomedical Research Ethics Committee (Vilnius, Lithuania), and the permission of the Ethics Committee to perform the study was given (08.07.2014 No.158200-14-743-260).

### 2.2. Ovarian Tissue Collection and Cryopreservation

One half or one third of an ovary was resected in the operating room of the Department of Oncogynecology of the NCI, and immediately transported on ice to the biobank of the NCI. The transporting medium consisted of 199 Earl’s medium (M199, Biochrom, Berlin, Germany) enriched with 5% *v/v* human serum albumin (HSA, OctaPharma, Manchester, UK), and 1% *v/v* penicillin/streptomycin/amphotericin B solution (Lonza, Houston, TX, USA).

Directly after the transportation to the biobank, the ovarian tissue was placed in 100 mm Petri dishes (Falcon, Corning, Tewksbury, MA, USA) with fresh transporting medium, and then cut into small pieces ~0.5 × 0.5 cm. Cryopreservation of the pieces was performed using slow freezing according to Herraiz et al.’s protocol [12]. The freezing medium consisted of M199 media enriched with 5% *v/v* HSA, 1% *v/v* penicillin/streptomycin/amphotericin B solution, and 10% *v/v* dimethylsulfoxide (Invitrogen, Canada). The cryoprotectant was added sequentially as described in Herraiz et al.’s protocol [12]. Tissue pieces were transferred to the cryoprotective solution in 2 mL cryotubes, placed in a Mr. Frosty Cryobox, transferred immediately to a –80 °C freezer (Arctiko, ULUF 450, Esbjerg, Denmark), and kept overnight. The next day, the cryotubes were placed in a –150 °C freezer (Sanyo Electric, Osaka, Japan) for long-term storage.

### 2.3. Ovarian Tissue Thawing and Xenotransplantation

**Figure 1:**
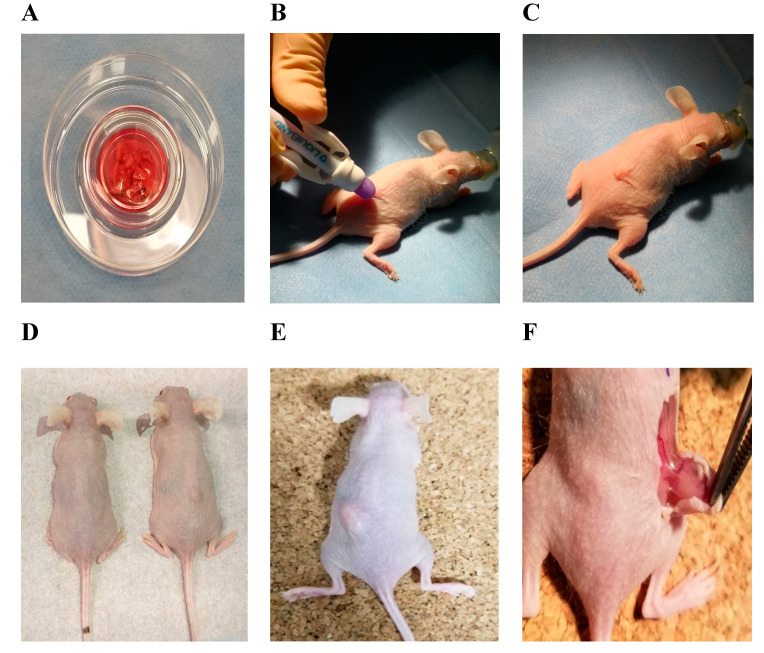
Representation of xenotransplantation experiment design into NOD.SCID hairless mice and the results. (**A**) Thawed ovarian tissue pieces containing cortex and medulla; (**B**) wound closure with liquid glue LiquiBand; (**C**) premedication and anesthesia by isoflurane gas; grafting of the ovarian tissue; (**D**) healthy control (left mouse) and engrafted frozen–thawed ovarian tissue fragment (right mouse) after 5 weeks; (**E**) formatted tumor 2 weeks after injection; (**F**) de novo vascularization in tumor after 2 weeks during follow-up.

After more than one year of storage, the ovarian tissue pieces, consisting of cortex and medulla, of five patients were thawed. Cryotubes with ovarian tissue pieces were removed from the ultra-low temperature freezer and transferred on ice. In the laboratory, cryotubes were immersed in a water bath at 37 °C for several minutes until the ice in the cryotubes started melting. The cryoprotective solution was diluted 1:1 with thawing solution and kept for 5 min at room temperature two times. A thawing solution consisted of M199 media supplemented with 5% *v/v* HSA and 1% *v/v* penicillin/streptomycin/amphotericin B solution. A small portion of each ovarian tissue sample was cut and used for the analysis of miRNA. The remaining ovarian tissue pieces (Figure 1A) were immediately submitted for xenotransplantation into immunodeficient NOD.SCID mice (Envigo, Wyton, UK).

Thirteen NOD.SCID female mice at 6 weeks of age were used for the xenotransplantation. The permission of the State Food and Veterinary Service to perform the experiment was given 23.04.2019 (No. G2–107), the maintenance and experimentation complied with the requirements stated in the 2010/63/EU Directive and Order of the Lithuanian State Food and Veterinary Service Director No. B1-866; 31-12-2012. Two weeks of acclimatization was given, animals were kept under sterile conditions, and had free access to sterile water and specific food (*Teklad Global 19% Protein Extruded Rodent Diet*, Envigo, Wyton, UK). Each mouse was given at least 70 cm^2^ of space in ventilated cages (in groups of four or five), in environmentally controlled housing with a light/dark cycle of 12/12 h, relative humidity of 50 (±4)% and a controlled temperature of 22 (±2) °C. Environmental enrichment included bedding (Tapvei Estonia OÜ, Paekna, Estonia) and sterilized paper for nesting. The surgery was performed in the afternoon. During the procedure, animals were separately anesthetized with 3% isoflurane gas (1000 mg/g) (Vetpharma Animal, Barcelona, Spain) (Figure 1B,C) in medical oxygen (0.6 L/min) using a single animal EZ–Anesthesia system EZ–SA800 and sedated by intramuscular injection of Sedin 1 mg/mL (Vetpharma Animal, Barcelona, Spain). After loss of the righting reflex, each mouse was transferred to a sterile surgical table with a surface temperature of 37 °C while inhaling the anesthetic via a nosecone. During the procedure of 5–10 min, the animals remained anesthetized with 1.5% of isoflurane gas in oxygen, and eyes were prevented from drying using Oftagel 2.5 mg/g (Santen OY, Osaka, Japan). The ovarian tissue pieces of cortex and medulla were grafted dorsolaterally near the vertebral column in each mouse (Figure 1C). The wound was lubricated with liquid glue LiquiBand (Advanced Medical Solutions, Winsford, UK). After completion of the procedure, the mice were placed in individual cages in a recovery area with thermal support until fully recovered. During housing, mice were monitored daily for health status.

Mice were divided into four groups: experimental group with grafted frozen–thawed ovarian tissue containing cortex and medulla (*n* = 5); 1st control group with grafted fresh ovarian tissue containing cortex and medulla (*n* = 5); 2nd control group with injected human breast cancer cells (MCF 7, *n* = 2); and a 3rd group of healthy controls (*n* = 1). Fresh ovarian tissue was taken in the operating room of the Department of Oncogynecology of the NCI on the same day of xenotransplantation and was transported directly in the transport medium to the Life Science Center (Vilnius, Lithuania). The cell line was provided by the Department of Biological Models cell bank (Life Sciences Center of Vilnius University, Lithuania). Animals from experimental, 1st control, and 3rd control groups were killed by cervical dislocation at the end of the 5th week and 2nd control group animals were sacrificed 2 weeks after xenotransplantation due to rapid growth of tumors and signs of distress; grafts (Figure 1D) and formatted tumors (Figure 1E,F) were recovered, put in formalin, and sent to the pathology laboratory for histological analysis.

### 2.4. Histological and Immunohistological Analysis

For the histological and immunohistochemical investigation, all tissue pieces (frozen–thawed and xenografted fresh and frozen–thawed tissues) were fixed in formalin solution, embedded in paraffin wax, and serially sectioned into 4 μm sections. Staining with hematoxylin/eosin and Ki–67 antibody was applied to all ovarian tissues (frozen–thawed and xenografted fresh and frozen–thawed tissues) and formatted tumor. The immunohistochemical analysis for Ki-67 was performed using an automated Roche Ventana Benchmark GX system (Ventana, Oro Valley, USA), using SP6 antibody (dilution 1:100; Code M3064, DAKO Agilent Technologies, Santa Clara, CA, USA). Ki-67 index was reported as the percentage of nuclear staining-positive cells (at least 1000). The percentage of positive staining was evaluated by experienced pathologists. All samples were analyzed under a light microscope (×40, ×100, ×200, Olympus, Tokyo, Japan) and evaluated by experienced pathologists at the Pathology Diagnostics Company (Vilnius, Lithuania).

### 2.5. RNA Isolation, Quantification, and Qualification

RNA from all five frozen–thawed ovarian fragments was extracted using a *miRNeasy Mini Kit* (Qiagen, Hilden, Germany) following the manufacturer’s instructions. Total RNA concentration and quality were measured by a NanoDrop 2000c spectrophotometer and verified by an Agilent 2100 Bioanalyzer using *RNA 6000 Nano LabChip* (Agilent Technologies, Santa Clara, CA, USA) according to the manufacturer’s recommendations.

### 2.6. MiRNA RT-qPCR Analysis

Three ovarian tissue samples with the best quality and quantity of extracted RNA were selected for miRNA level analysis with *miRNome miScript miRNA PCR Array #6* (MIHS-216Z, SABiosciences, Qiagen). Template complementary DNA (cDNA) was synthesized from total RNA with a *miScript II RT Kit* and *miScript HiSpec Buffer* (Qiagen, Hilden, Germany) following the manufacturer’s protocol. The templates were mixed with *RT^2^ SYBR Green qPCR Master Mix* (Qiagen, Hilden, Germany) and 20 μL were aliquoted into each well for the RT-qPCR array. An array consisted of a panel of 96 primer sets of 84 unique miRNAs of interest, two *C*eanorhabditis *elegans* miR-39, and 10 controls. RT-qPCR was performed in a Rotor-Gene Q thermocycler (Qiagen, Hilden, Germany), as follows: 15 min at 95° C and 40 cycles of 15 sec at 94° C, 30 sec at 55° C, and 30 sec at 70° C. Each sample was tested in technical duplicate. Each biological and technical replicate was tested in a new array. The primary miRNA data were analyzed using online software *miScript miRNA PCR Array Data Analysis*. The relative expression of each target miRNA was determined with the ΔΔC_t_ method and normalized to the geometric mean of six small RNAs (SNORD61, SNORD68, SNORD72, SNORD95, SNORD96A, and RNU6-2) according to the SABioscience guide.

### 2.7. Prediction and Analysis of Target Genes of Highly Expressed miRNAs

The target genes of highly expressed miRNAs were predicted using miRDB (www.mirdb.org) [23]. Our chosen prediction score was >89. A protein–protein interaction (PPI) network of target genes was constructed using STRING database v11 (www.string-db.org) [24] and the target genes with the largest degree of connectivity were identified by using Cytoscape software v3.6.1 (the *NetworkAnalyzer* option was used to compute the degree and *cytoHubba* was used to visualize the most connected targets) [25]. Targets with the highest degree then were analyzed by the Database for Annotation, Visualization, and Integrated Discovery (DAVID, www.david.abcc.ncifcrf.gov) online program to understand the biological meaning behind targeted genes [26]. DAVID was used for gene ontology (GO) functional annotation, using *p*-value <0.05.

## 3. Results

### 3.1. Characteristics of Patients 

Twenty-eight young female cancer patients from the NCI, Vilnius, Lithuania were included in the “*Fertility preservation program for cancer patients*”. The women’s mean age was 32.4 years (the youngest 26 y/o, the oldest 39 y/o). According to the diagnosis, 53% of the women were diagnosed with breast cancer, 21%—ovarian, 18%—cervical cancer. One woman was diagnosed with rectal cancer and one women with B cell lymphoma. The personal and clinical characteristics of the women are presented in Table 1. 

### 3.2. In Vivo Study

#### 3.2.1. Graft Recovery Rate and Macroscopic Evaluation

Macroscopic analysis showed great results of engraftment. In the experimental group with frozen-thawed ovarian tissue grafts, four out of five tissue pieces (80%) engrafted successfully (Figure 1D). In the 1st control group, five fresh ovarian tissues were grafted. One mouse scraped out the tissue the next day. In the remaining four mice, all four tissue pieces (100%) were engrafted successfully. In the 2nd control group, tumor formation in both mice was visible after 2 weeks (Figure 1E,F). It was observed that all engrafted tissues had some vessels supporting them. The mice of the negative control (3rd group) stayed healthy without any changes or spontaneous tumor formation during the experiment.

#### 3.2.2. Histological and Immunohistochemical Analysis of Xenografted Tissue

**Figure 2:**
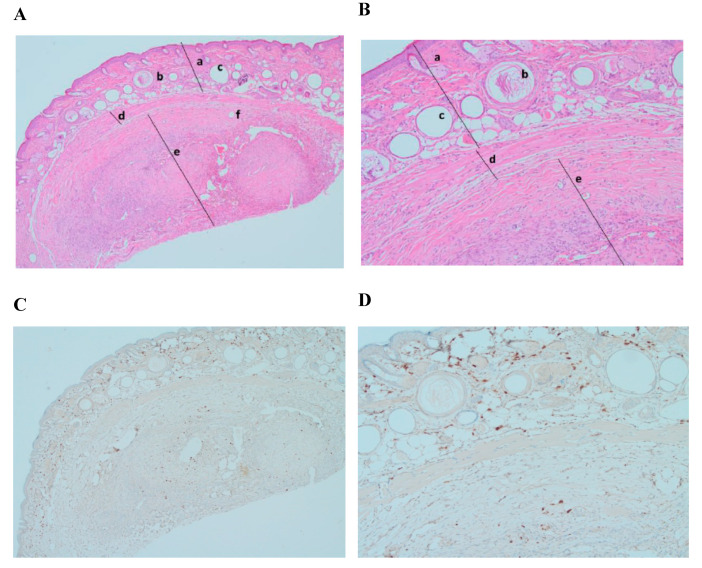
Histological and immunohistochemical analysis of frozen–thawed ovarian tissue graft. Hematoxylin/eosin staining (**A**) (magnification ×40) and (**B**) (magnification ×100): (a) NOD.SCID mouse skin; (b) hair follicles; (c) fat follicles; (d) intermediate zone—de novo formatted connective tissue with neovascularization; (e) human ovarian tissue; (f) developing follicle. Ki–67 staining (**C**) (magnification ×40) and (**D**) (magnification ×100): no malignant transformation showed (expression < 5%).

Histological analysis demonstrated a significant engraftment of frozen–thawed tissue samples (Figure 2A,B, mark d). Hematoxylin/eosin-stained slides showed formation of a new intermediate zone and elements of neovascularization between mouse skin and the graft (Figure 2A,B, mark d). Ki–67 immunostaining did not show any formation or presence of cancer cells in xenografted ovarian tissue (expression of Ki–67 < 5%) [27] (Figure 2C,D). 

**Figure 3:**
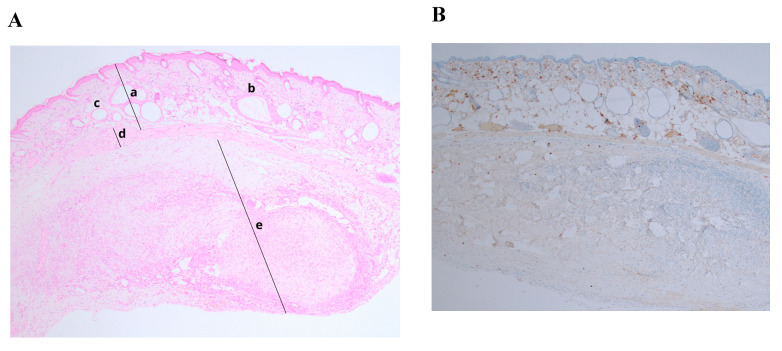
Histological and immunohistochemical analysis of fresh ovarian tissue graft. (**A**) Hematoxylin/eosin staining (magnification ×40): (a) NOD.SCID mouse skin; (b) hair follicles; (c) fat follicles; (d) intermediate zone—de novo formatted connective tissue with neovascularization; (e) human ovarian tissue; (**B**) Ki–67 staining, expression < 5% (magnification ×40).

Histology of xenotransplanted fresh tissue controls (1st control) demonstrated successful engraftment after 5 weeks. Stained slides showed an intermediate zone and neovascularization as in the experimental group. Ki–67 staining showed no abnormal proliferation rate (Ki–67 expression < 5%) (Figure 3B). There were no visible differences between engraftment of frozen–thawed or fresh ovarian tissue.

**Figure 4:**
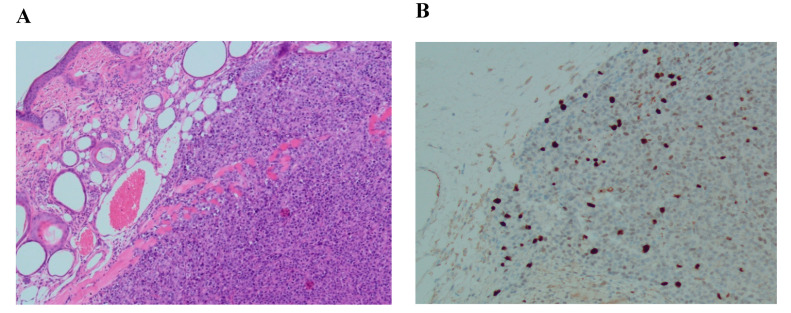
Histological and immunohistochemical analysis of formatted tissue of MCF7 line. (**A**) Hematoxylin/eosin staining (magnification ×100); (**B**) Ki–67 staining, expression > 10% (magnification ×100).

Histological analysis of positive controls (2nd control) demonstrated successful cancerous tissue formation 2 weeks after cell injection. The results of injected human breast cancer cells showed poorly differentiated and proliferating cells. Ki–67 staining showed an abnormal proliferation rate (Ki–67 expression > 10%) (Figure 4B).

### 3.3. In Vitro Study

#### 3.3.1. Histology and Immunohistochemical Analysis of Frozen–Thawed Ovarian Tissue

**Figure 5:**
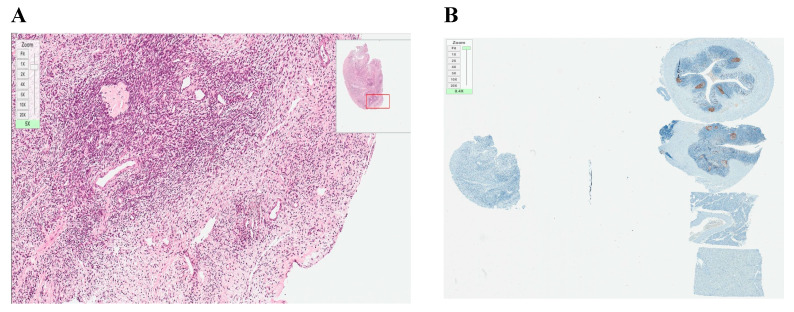
Histological and immunohistochemical analysis of frozen–thawed ovarian tissue. (**A**) hematoxylin/eosin staining (magnification ×20); (**B**) Ki–67 staining, no malignant transformation (expression < 5%) (magnification ×20). Both pictures originally magnified ×20 but the zoom scale in the upper left corner of each picture specifies additional zooming.

To be able to compare the efficacy of identifying the existence of malignant cells in xenografted and only frozen–thawed ovarian tissue samples, analogous histological and immunostaining assessments were performed. Analyzed frozen–thawed ovarian tissue fragments showed no evidence of structural damage or overproliferation (Ki–67 expression < 5%) (Figure 5).

#### 3.3.2. Total RNA Extraction, Quantity and Quality Assessment of Frozen–Thawed Ovarian Tissue

For further investigation, the quality and quantity of the total RNA were evaluated. The quality and quantity controls were performed by a UV spectrophotometer and Agilent 2100 bioanalyzer. As shown in Table 2, total RNA isolated from all samples was pure as indicated by the *A_260_/_280_* ratio, of sufficient integrity as reflected by the RNA integrity number (RIN), and of good concentration.

#### 3.3.3. Molecular Analysis of Frozen–Thawed Ovarian Tissue

**Figure 6:**
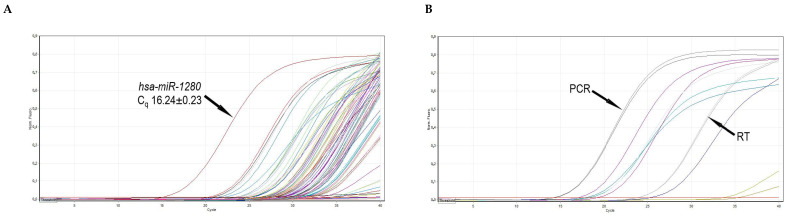
Representative RT-qPCR amplification curves. (**A**) Amplification curves of 84 miRNA cDNAs of frozen–thawed ovarian tissues. The highest expression of hsa-miR-1280 is indicated by an arrow. (**B**) Amplification curves of two RT, two PCR (indicated), and six small RNA controls. C_q_—quantification cycle; RT—reverse transcription; PCR—polymerase chain reaction.

To evaluate the profiles of miRNA in frozen–thawed ovarian tissue, we measured the expression of 84 well-characterized miRNAs in the human miRNA genome using a commercial RT-qPCR array. Raw data quality control performed using the manufacturer’s online software *miScript miRNA PCR Array Data Analysis* confirmed that reverse transcription and polymerase chain reaction were efficient (Figure 6B).

Altogether, the expression of 80 miRNAs (95% of all investigated miRNAs) was detected (C_q_ < 33). Four miRNAs were not expressed (C_q_ ≥33) in thawed ovarian tissue samples. Moreover, fifteen miRNAs were expressed at a high level (C_q_ < 25) with the highest expression (C_q_ 16.24 ± 0.23) detected for *hsa-miR-1280* (Figure 6A), and these miRNAs constituted 18% of all the evaluated miRNAs (Table 3). The remaining 65 miRNAs had intermediate or low expression levels.

#### 3.3.4. Target Prediction and Functional Annotation Analysis

**Figure 7:**
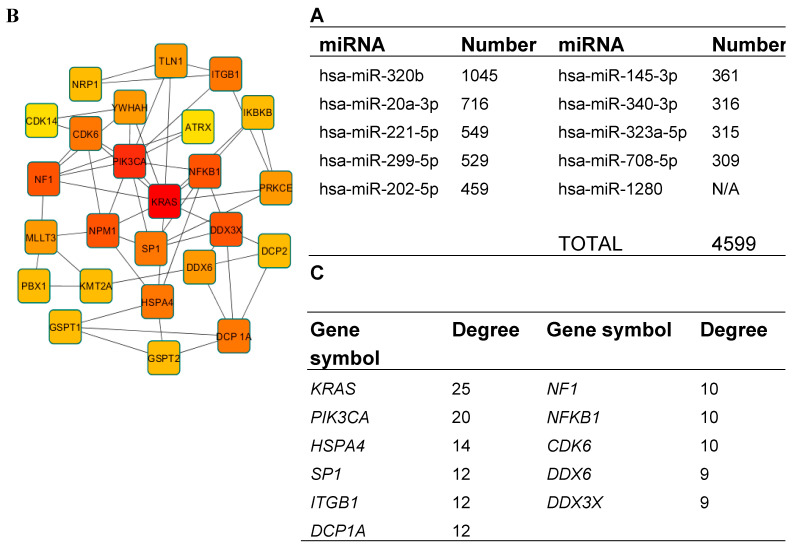
Target prediction and protein–protein interaction (PPI) analysis. (**A**) The predicted target number of the 10 most highly expressed miRNAs. For miRNA–1280, no targets were detected; (**B**) the top 25 genes in PPI network of predicted target genes (node color: deeper colors indicate higher degree); (**C**) the top 11 genes identified in PPI network with the highest degree of connectivity.

In order to better understand the underlying biological processes of miRNAs, we performed target gene prediction and enrichment analysis of the 10 most abundantly expressed miRNAs: hsa-miR-1280, hsa-miR-320b, hsa-miR-20a-3p, hsa-miR-221-5p, hsa-miR-299-5p, hsa-miR-202-5p, hsa-miR-145-3p, hsa-miR-340-3p, hsa-miR-323a-5p, hsa-miR-708-5p (Figure 7A). 

However, no results were found for *hsa-miR-1280* on any miRNA target prediction databases. Nine out of 10 miRNA targets were successfully predicted by miRDB. The results are shown in Figure 7A. In total, 4599 target genes were predicted and miRNA–320b was found to potentially target the most genes—1045. For further analysis, only targets with a target score equal to or greater than 90 were chosen—in total, 339. 

**Figure 8:**
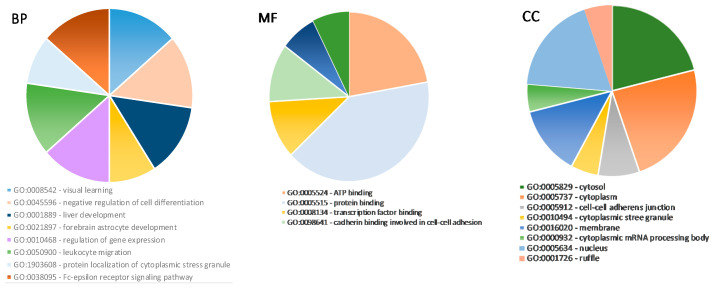
Gene ontology analysis for predicted target genes of miRNAs. Top enriched biological processes (BP) (**left graphic**); top enriched molecular functions (MF) (**middle graphic**); top enriched cellular components (CC) (**right graphic**) for predicted target genes.

Then, PPI data were visualized using Cytoscape software to determine the degree of connectivity; the top 11 genes, *KRAS, PIK3CA, HSPA4, SP1, ITGB1, DCP1A, NF1, NFKB1, CDK6, DDX6,* and *DDX3X* (Figure 7B,C), were identified and used for further analysis. Identified target genes were analyzed using DAVID for GO functional annotation for all three categories: cellular component, molecular function, and biological process (Figure 8).

## 4. Discussion

This pilot study has shown a couple of relevant findings: (1) the slow-freezing technique is a reliable method for OTC; (2) the first successful xenotransplantation of human frozen–thawed or fresh ovarian tissue in Lithuania; (3) histology, immunohistochemistry, and molecular analysis could be sufficient methods for quality control to verify the viability of frozen–thawed ovarian tissue and the risk level of metastatic disease. Not to forget that OT transplantation is not only for preserving fertility, it also restores endocrine function in cancer survivors [28].

It is possible to determine the risk if stored ovarian tissue contains malignant cells by using a murine xenotransplantation model. If the xenotransplantation experiment shows no evidence of contamination, the chance of reintroducing cancer cells is considered low. One of our study limitations could be a small sample size of individuals in in vivo and in vitro experiments. However, the xenotransplantation method concerns animal rights [29] and takes more time. Nonetheless, the number of animals used in each group was enough to get reliable results following ethical requirements and principles of reducing, replacing, and refining animals in experimentation. For this reason, we have performed our study to assess if in vitro quality control methods, e.g., histology, immunohistochemistry, and RT–qPCR, on the same patients’ samples could replace the in vivo technique.

The results demonstrated that 5 weeks after xenotransplantation into immunodeficient mice, the slow-freezing cryopreservation protocol we used allowed for the conservation of a high quality tissue morphology of ovarian tissue (Figure 2) compared to the control of xenografted fresh ovarian tissue (Figure 3) or only frozen–thawed ovarian tissue (Figure 5). Eighty percent engraftment efficiency of frozen–thawed ovarian tissue was established, and in the remaining four engrafted fragments, the neovascularization process was visible. Blood supply is crucial for the successful engraftment of tissue and the survival of ovarian follicles [30,31]. Moreover, in some slides from the histological evaluation of frozen–thawed ovarian tissue, morphologically intact follicles were found without any additional ovarian stimulation. These results are in line with some studies performed by other investigators where the survival of follicles and efficacy of different OTC protocols were evaluated [11,12,14]. However, researchers are looking for more reliable methods to evaluate follicles, and one group of researchers has recently proposed a simple method to quantify only viable follicles by neutral red dye since histological evaluation with hematoxylin/eosin shows all the follicles [32]. Nonetheless, for accuracy, our results were validated by injection with cancerous cells and rapid tumor formation in the mice of the positive control group. In addition, our study provides evidence of a reliable methodology for ovarian tissue freezing for at least 4 years and thawing without any noticeable loss of tissue viability.

We were trying to evaluate a diagnostic tool based on histological and immunohistochemical staining alongside a molecular method to evaluate the quality of frozen–thawed ovarian tissue of cancer patients who underwent procedures of fertility preservation. Histological analysis demonstrated an intact structure and no evidence of disease in frozen–thawed ovarian tissue (Figure 5). However, to validate or deny the presence of malignant cells by microscopy is not sufficient for some types of cancer [33]. Our results showed that isolated RNA from conventionally frozen–thawed ovarian tissues was pure and sufficiently intact for further experiments. This could demonstrate that the slow-freezing cryopreservation protocol is indeed effective in terms of keeping the stability of genes. A previous study of Isachenko and others [16] has provided some evidence of higher RNA degradation after vitrification in comparison with the slow-freezing technique, which once more supports our chosen cryopreservation method. 

Many studies have been conducted to search for cancer biomarkers using mRNA from cryopreserved ovarian tissue or section staining of the tissue with antibodies [16,18,34,35]. Different research groups have started conducting studies to search for circulating miRNAs [36,37] or miRNAs in the tissue [20,38,39,40] as specific cancer biomarkers, but a study of miRNA expression after cryopreservation has just been recently published [19]. This study showed that upregulated miRNA–24 and downregulated expression of miRNA–193b, miRNA–320A, and steroid production could be non-invasive (expression patterns in culture media) markers for cell damage. One group of scientists has investigated miRNA expression in mice ovaries during exposure to chemotherapy and how it is related to follicle apoptosis and depletion afterwards [41]. They have found that the downregulation of the highly conserved let–7a miRNA is an indication of chemotherapy-induced ovarian injury, and its restoration can protect early stage follicles from the damage. Other scientific groups have shown the prevention of follicle atresia in vivo and inhibited apoptosis in vitro by exosome-derived miR–10a [42] and restoration of ovarian function impairment by miR–21 after chemotherapy [10]. These examples show why the investigation of miRNAs has been brought not only into the field of cancer treatment but female fertility protection as well.

One of the tasks of our investigation was to evaluate if miRNA expression from frozen–thawed ovarian tissue would be sufficient for detection. However, there are not many studies that investigate miRNAs related to the survival of follicles, development, and sustained damage after thawing the tissue. For the majority of scientists, the field of interest is miRNAs involved in cancer, epithelial mesenchymal transition, or angiogenesis. Hence, another limitation of this study is that we could not perform miRNA analysis on a control sample, e.g., ovarian cancer tissue or fresh ovarian tissue, and we are not able to state if the expression is elevated or downregulated. Though our original thought was to compare expression profiles of miRNAs in frozen–thawed ovarian tissue and in vitro matured ovarian tissue after cryopreservation, the results of RNA quality after in vitro maturation were poor and we could not perform RT-qPCR on those samples (data not provided). Our results could only be predicted by the previously conducted studies or in silico analysis. For example, in the study of investigating biomarkers for ovarian cancer, one of the miRNAs is miR–135b–3p and its expression should be upregulated in the case of human epithelial ovarian cancer [40]. Therefore, in our analysis, expression was detected as low (Table 3, 30 ≤ C_q_ < 33). Hsa–miR–708 in our study showed high expression and even though we cannot claim it is actually higher than in cancerous tissue, this could be in line with not being downregulated in the case of ovarian cancer metastasis [43]. Members (miR–200a/b/c, –141, and –429) of the miR–200 family are considered as reliable markers of epithelial ovarian cancer when overexpressed [39]. Nevertheless, the upregulation of miR–200b was demonstrated in granulosa cells of polycystic ovary syndrome and was able to suppress proliferation of granulosa-like tumor cell lines [22]. Another miRNA involved in many processes is miR–145. It participates in primordial follicle development and maintenance in mice [44], has higher expression levels in the follicular stage than the luteal stage in sheep experiments [45], or suppresses colorectal cancer cell viability and proliferation [46], but when downregulated, it enhances oocyte maturation and cell survival [47] or might serve as an ovarian cancer biomarker [39,48]. MiR–146 is reported to control reproductive functions by inhibiting the release of ovarian progesterone [49], whereas the downregulation of miR–146b–3p expression suppresses the proliferation and migration of cervical cancer cells [50]. Family members of miR–34 are involved in the development in both sexes of bovine gametes and embryos, but primary miR–34b/c is only detected in primordial oocytes and reproductive tissue [22]. The earliest expression was detected for hsa–miR–1280 (Figure 7). It is a non–conserved miRNA whose expression is upregulated or downregulated depending on the type of tumor [51,52,53]. Nonetheless, it has been shown to be involved in embryo development [51]. Xu and colleagues have investigated different miRNA expression levels during oocyte development and found that hsa–miR-20a expression in germinal vesicle stage oocytes was higher (29.48 ± 0.11) compared with in vitro matured MII stage oocytes (33.46 ± 0.22) [54]. The same group of scientists found expression of hsa–miR–376a, hsa–miR–297, and hsa–miR–299–5p in both stages. MicroRNA–376a overexpression was also found to increase primordial follicles and reduce follicle apoptosis in mice [55]. MiR–513a–3p, miR–513b–5p, and miR–513c–5p are detectable in normal human granulosa cells [56,57]. Our future studies will involve broader analysis of the miRNA profile after cryopreservation, and comparison with cancerous tissue in order to evaluate the malignancy of the tissue samples.

Gene ontology analysis showed that predicted gene targets are involved in ATP, protein, and transcription factor binding, regulation of gene expression, negative regulation of cell differentiation or liver development, and are also distributed in different cell compartments: cytoplasm, membrane, and nucleus. However, at present, we have not found any studies on GO analysis and various miRNA impacts on the tissue vitality or viability of thawed ovarian tissue. These primary results need further investigation by including more biological samples and various techniques (like in vitro maturation of human oocytes).

The results showed no evidence for viable malignant cells in any case—neither in the xenotransplantation model nor in the staining panels. The main limitation of histological and molecular assessments is that the assay is performed on another or a small piece of frozen–thawed ovarian tissue, and hence the piece is destroyed and not used for the autotransplantation procedure. Moreover, detection of only tumor-specific markers could be misleading due to belonging to non–viable cancer cells in the ovarian tissue [58,59]. A complementary use of other detection methods for possibly malignant cells is important, such as cytometry, genetic tests, or, as in this study—histology and immunostaining.

## 5. Conclusions

Our study demonstrates an efficient way to cryopreserve ovarian tissue prior to cancer treatment for future restoration of reproductive functions for cancer survivors. Ovarian tissue cryopreservation technology was successfully implemented in a practical use for cancer patients at the National Cancer Institute (Vilnius, Lithuania) and Vilnius University Hospital Santaros Klinikos (Vilnius, Lithuania). We have shown that xenotransplantation could be potentially replaced by more recent histological and molecular methods. Molecular methods have shown that miRNAs might be potentially used as identifiers of healthy/diseased tissue after further analysis. However, the possibility of reintroducing malignant cells remains despite all the achievements in OTC development and transplantation. Newer, safer, and more specific approaches need to be developed in order to eliminate the risk of reoccurrence of the disease. Future studies should involve further molecular analysis, tissue or oocyte maturation in vitro, and artificial ovary development, while minimizing animal use for quality control experiments.

## Figures and Tables

**Table 1:** Individual and clinical women’s characteristics. TNM—tumor, nodes, and metastasis category system; BT—borderline tumor.

Code	Age	Date of Tissue Cryopreservation	Cancer Site	Histology	TNM Staging	G (Grade)
VIP-M-0001	29	2014.10.20	Ovary	Adenocarcinoma	pT3AN1M0(BT)R0	BT
VIP-M-0002	25	2014.11.20	Ovary	Disgerminoma	pT2aN0M0	-
VIP-M-0003	37	2015.01.26	Cervix uteri	Squamous cell carcinoma	pT2bN0M0G3R0	G3
VIP-M-0004	27	2015.03.23	Ovary	Adenocarcinoma	pT1CN0M0G1R0	G1
VIP-M-0005	39	2015.06.09	Cervix uteri	Squamous cell carcinoma	cT3bN1M0G3	G3
VIP-M-0006	31	2015.07.27	Cervix uteri	Squamous cell carcinoma	pT1b1N0M0G3R0	G3
VIP-M-0007	32	2015.11.25	Cervix uteri	Adenocarcinoma	pT1bN0M0G3L1V1R0	G3
VIP-M-0008	27	2015.11.25	Rectum	Adenocarcinoma	cT3N2M0G1	G1
VIP-M-0010	39	2016.02.10	Ovary	Mucinal borderline Ca	pT1CN0M0G(BT)R0	BT
VIP-M-0011	28	2016.03.29	Breast	Invasive ductal Ca	cT2N0M0	G3
VIP-M-0012	26	2016.04.21	Ovary	Serosal cystadenoma	pT1CNXM0G(BT)R0	BT
VIP-M-0013	38	2016.05.04	Breast	Invasive ductal Ca	cTxN1M0	G2
VIP-M-0014	37	2016.06.09	Breast	Invasive ductal Ca	cT1N1M0	G3
VIP-M-0015	39	2016.08.11	Breast	Invasive ductal Ca	pT2N1M1	G3
VIP-M-0016	35	2016.10.24	Breast	Invasive ductal Ca	pT1cN0M0	G2
VIP-M-0017	28	2016.11.14	Lymphoma	B cell	-	-
VIP-M-0018	21	2016.11.25	Ovary	Serosal borderline Ca	pT1CNX0G(BT)R0	BT
VIP-M-0019	36	2016.12.27	Cervix uteri	Squamous cell carcinoma	cT2aN0M0	G3
VIP-M-0020	35	2016.12.27	Breast	Invasive ductal Ca	cT3N2M0	G2
VIP-M-0021	40	2017.04.24	Breast	Invasive ductal Ca	pT1bN0(sn)M0	G2
VIP-M-0022	36	2017.06.07	Breast	Invasive ductal Ca	cT1CN1M0	G3
VIP-M-0023	29	2017.08.24	Breast	Invasive ductal Ca	Unknown	-
VIP-M-0024	28	2017.10.06	Breast	Invasive ductal Ca	pT2N0(SN)M0	G2
VIP-M-0025	27	2017.12.08	Breast	Invasive ductal Ca	pT1cN0(sn)M0	G2
VIP-M-0026	38	2018.03.07	Breast	Invasive ductal Ca	cT3N1M0	G2
VIP-M-0027	31	2018.07.09	Breast	Adenocarcinoma	pT2N0(sn)M0	G3
VIP-M-0028	34	2018.08.22	Breast	Invasive ductal Ca	cT2N1M0	G3
VIP-M-0029	36	2018.10.24	Breast	Invasive ductal Ca	c.T3N3M0	G3

**Table 2:** Quality and quantity indicators of extracted RNA from frozen–thawed ovarian tissues. RIN—RNA integrity number; RT-qPCR—reverse transcription quantitative polymerase chain reaction.

Indicator	Expected Value for RT-qPCR	Samples				
		*VIP–M-006*	*VIP–M-007*	*VIP–M-008*	*VIP–M-011*	*VIP–M-012*
***Concentration (NanoDrop)***	>21 ng/μL	474	226	401	41	177
***Concentration (Agilent)***		343	145	257	23	120
***A_260_/A_280_ (NanoDrop)***	1.9–2.1	2.00	2.04	2.02	1.96	2.06
***RIN (Agilent)***	>6.5	6.80	6.60	7.20	7.60	7.70

**Table 3:** Expression levels of miRNAs investigated in frozen–thawed ovarian tissue samples (VIP–M-006, VIP–M-008, and VIP–M-012 samples were analyzed). C_q_—quantification cycle.

Expression Level	C_q_ Interval	miRNAs
High	C_q_ < 25	hsa-miR-145-3p, hsa-miR-202-5p, hsa-miR-20a-3p, hsa-miR-221-5p, hsa-miR-299-5p, hsa-miR-320b, hsa-miR-323a-5p, hsa-miR-340-3p, hsa-miR-1280, hsa-miR-708-5p, hsa-miR-628-5p, hsa-miR-513b-5p, hsa-miR-513c-5p, hsa-miR-1180-3p, hsa-miR-342-5p
Intermediate	25 ≤ C_q_ < 30	hsa-miR-378a-5p, hsa-miR-132-5p, hsa-miR-485-5p, hsa-miR-99b-3p, hsa-miR-320d, hsa-miR-1203, hsa-miR-1299, hsa-miR-539-5p, hsa-miR-510-5p, hsa-miR-30b-3p, hsa-miR-376a-5p, hsa-miR-146b-3p, hsa-miR-1285-3p, hsa-miR-1301-3p, hsa-miR-708-3p, hsa-miR-513a-3p, hsa-miR-513c-3p, hsa-miR-1290, hsa-miR-1908-5p, hsa-miR-219a-1-3p, hsa-miR-1179, hsa-miR-1207-5p, hsa-miR-200b-5p, hsa-miR-34b-3p, hsa-miR-432-3p, hsa-miR-1287-5p, hsa-miR-1303, hsa-miR-191-3p, hsa-miR-454-5p, hsa-miR-1226-3p, hsa-miR-1208, hsa-miR-380-5p, hsa-miR-1322, hsa-miR-1286, hsa-miR-377-5p, hsa-miR-1185-5p, hsa-miR-1283, hsa-miR-1224-5p, hsa-miR-1184, hsa-miR-488-5p, hsa-miR-1183, hsa-miR-34b-5p, hsa-miR-452-3p
Low	30 ≤ C_q_ < 33	hsa-miR-1289, hsa-miR-570-3p, hsa-miR-302b-5p, hsa-miR-1288-3p, hsa-miR-1302, hsa-miR-297, hsa-miR-363-5p, hsa-miR-491-3p, hsa-miR-1321, hsa-miR-1323, hsa-miR-875-5p, hsa-miR-1278, hsa-miR-1206, hsa-miR-1182, hsa-miR-135b-3p, hsa-miR-1228-5p, hsa-miR-449b-5p, hsa-miR-1205, hsa-miR-561-3p, hsa-miR-519d-3p, hsa-miR-1207-3p, hsa-miR-519e-3p, hsa-miR-1204
Not expressed	C_q_ ≥ 33	hsa-miR-1284, hsa-miR-526b-3p, hsa-miR-1178-3p, hsa-miR-1279
